# Conserved and species-specific alternative splicing in mammalian genomes

**DOI:** 10.1186/1471-2148-7-249

**Published:** 2007-12-22

**Authors:** Ramil N Nurtdinov, Alexey D Neverov, Alexander V Favorov, Andrey A Mironov, Mikhail S Gelfand

**Affiliations:** 1 Faculty of Bioengineering and Bioinformatics, M.V. Lomonosov Moscow State University, Vorbyevy Gory 1-73, Moscow, 119992, Russia; 2 State Research Institute for Genetics and Selection of Industrial Microorganisms "GosNIIGenetika", 1st Dorozhny proezd 1, Moscow, 117545, Russia; 3 Division of Oncology Biostatistics and Bioinformatics, The Sidney Kimmel Cancer Center at Johns Hopkins, 550 North Broadway, Suite 1103, Baltimore, MD 21205, USA; 4 Institute for Information Transmission Problems, Russian Academy of Sciences, Bolshoi Karenty pereulok 19, Moscow, 127994, Russia

## Abstract

**Background:**

Alternative splicing has been shown to be one of the major evolutionary mechanisms for protein diversification and proteome expansion, since a considerable fraction of alternative splicing events appears to be species- or lineage-specific. However, most studies were restricted to the analysis of cassette exons in pairs of genomes and did not analyze functionality of the alternative variants.

**Results:**

We analyzed conservation of human alternative splice sites and cassette exons in the mouse and dog genomes. Alternative exons, especially minor-isofom ones, were shown to be less conserved than constitutive exons. Frame-shifting alternatives in the protein-coding regions are less conserved than frame-preserving ones. Similarly, the conservation of alternative sites is highest for evenly used alternatives, and higher when the distance between the sites is divisible by three. The rate of alternative-exon and site loss in mouse is slightly higher than in dog, consistent with faster evolution of the former. The evolutionary dynamics of alternative sites was shown to be consistent with the model of random activation of cryptic sites.

**Conclusion:**

Consistent with other studies, our results show that minor cassette exons are less conserved than major-alternative and constitutive exons. However, our study provides evidence that this is caused not only by exon birth, but also lineage-specific loss of alternative exons and sites, and it depends on exon functionality.

## Background

Alternative splicing is emerging as one of the major evolutionary mechanisms for protein diversification and proteome expansion. Indeed, not only more than half of mammalian genes are alternatively spliced [1-3], but a considerable fraction of alternative splicing events appears to be species- or lineage-specific, at the level of comparison of genes from human and mouse [4-7], rodents [8] or other mammals [9,10]; fruit flies (Drosophila melanogaster and D. pseudoobscura) and malarial mosquito [11]; or rice and Arabidopsis [12].

However, the prevalence and functionality of non-conserved alternatives is subject to controversy. Indeed, non-conserved cassette exons are often frame-shifting or contain in-frame stop codons, and thus their inclusion leads to isoforms likely subject to nonsense-mediated decay (NMD) [13]. On the other hand, this does not necessarily mean that such isoforms are devoid of function: channeling a transcript to NMD may be one of the regulatory mechanisms [14,15]. Functionality of minor isoforms is supported by the fact that many of them are tissue-specific [5], although in a study that used oligonucleotide microarrays, NMD-inducing isoforms have been shown to be expressed at uniform, low level [16].

In any case, pairwise comparisons do not allow one to distinguish between gain and loss of features such as splicing alternatives. Two recent studies that considered more than two genomes [5,11] just listed the estimates obtained in independent pairwise comparisons. In a study with triple human-mouse-rat comparison, about 20% of exons conserved in human and one rodent were not conserved in the other rodent [17], although this result could be biased by the procedure that used cross-species EST-to-genome alignments. Multiple genome analyses [9,10] considered progressively distant genome triples and demonstrated relatively recent gain of human minor isoform exons.

Here we compiled a set of human-mouse-dog ortholog triples and studied the conservation of human alternative splicing patterns in the mouse and dog genomes. In such comparisons, dog serves as an outgroup. Thus we can distinguish between gain and loss of a human alternative exon or site, although it still is not clear whether a gained alternative variant is functional or represents splicing noise (to distinguish between bona fide gains and noise using only evolutionary considerations, one has to consider gains that had occurred in internal branches of the phylogenetic tree and were conserved after that). In an attempt to address the functionality issue, we considered separately major (mostly included) and minor (mostly skipped) cassette exons, alternative splice sites corresponding to shorter or longer exon variants (internal and external alternative splice sites, respectively), and frame-preserving or frame-shifting alternatives. We also demonstrate that the observed distribution of minor (rarely used) internal and external splice sites is consistent with the model of random functional fixation of cryptic sites.

## Results

### Data compilation and preparation

Available ESTs, mRNA and protein sequences were mapped to the human genome. Unspliced or badly aligned ESTs were ignored. Of 20809 genes in the initial sample, 19669 genes had introns, and of the latter, 12595 genes had at least one splicing alternative. The alternatives were decomposed into 34463 elementary alternatives of the four main types: alternative donor and acceptor sites, cassette exons and retained introns. The former three types (alternative sites and cassette exons) occurring within protein-coding genes were considered in detail. After application of all filters described in the "Data and Methods" section, the final sample consisted of 18910 elementary alternatives.

Triples of orthologous human, mouse and dog genes were taken from [18]. A human exon was assumed to be conserved in the mouse or dog genome if spliced alignment of the genomic fragment containing this exon and adjacent exons on both sides yielded exactly the same exon triple. An alternative splice site was assumed to be conserved if invariant dinucleotides (GU for donor sites and AG for alternative sites) of both alternative sites were conserved. Note that (i) only the theoretical possibility of the conserved-exon existence is thus demonstrated, whereas its functional relevance could not be assessed, (ii) this approach allowed for the analysis of human genome-specific alternative exons and sites having no counterparts in the mouse and dog genomes, but not exons that are alternatively spliced in the human genome, but constitutively spliced in these genomes [7], and that (iii) absence of the exon or site in the sample of mouse or dog ESTs does not influence this definition. Thus the level of coverage of the mouse and dog genomes by the ESTs did not affect the results.

The major human variant was assumed to be the one that was observed in a protein and had the larger EST coverage. At that, the second variant was allowed to be supported only by ESTs. Cases where both variants were supported only by ESTs, as well as rare cases where the single protein-defined variant had lower EST support than the alternative variant were filtered out.

To compute the inclusion level of a human cassette exon, we considered all valid ESTs whose spliced alignment to the genome contained, at least partially, both adjacent exons. The inclusion level was defined as the fraction of the number of sequence fragments containing this exon to the total number of fragments covering this region. Note, however, that since an average EST is rather short, this procedure may discriminate against exon inclusion events, and thus their prevalence may be underestimated.

Similarly, to estimate the prevalence of an alternative site, spliced alignment of ESTs containing (at least partially) the exon spliced at this site and the adjacent exon was considered. Rare exons and sites that could arise from splicing errors were defined using the procedure from [19]. In a nutshell, a variant was considered "rare" (and hence suspicious), if the hypothesis that its frequency is less than 1% could not be rejected at 95% significance level given the observed counts of variants of the considered elementary alternative (see "Data and Methods").

Finally, all alternatives were divided in two groups, frame-preserving ones where the length of the alternative region was a multiple of three, and frame-shifting ones. Since we considered only protein-coding regions, no in-frame stop codons were allowed.

### Conservation of alternative exons and splice sites

We tested conservation of all observed human exons in genomic DNA of corresponding orthologous mouse and dog genes using a two-step procedure (see "Data and Methods"). To validate this procedure, we calculated conservation of constitutively spliced internal exons at varying levels of ESTs coverage (Table 1). These results agree with previously published estimates of 93–98% in [20]. The degree of conservation of constitutively spliced exons increases to 100% with increased EST coverage. Further, since we aligned human exons to genomic DNA using spliced alignment of exon chains, only exons that evolve considerably faster than adjacent exons so that they cannot be identified any more in the specific intron by a sensitive dynamic programming algorithm could escape detection. We consider such possibility rather unlikely.

**Table 1:** Conservation of constitutively spliced human internal exons

	**Conservation:**		**Conserved exons in:**			
	
**EST coverage**	**Mouse**	**Dog**	**Both mouse and dog**	**Mouse only**	**Dog only**	**Neither mouse nor dog**
**0 ESTs**	96.9%	97.0%	3042	65	69	32
**1 and more ESTs**	97.4%	97.9%	21318	290	421	165
**10 and more ESTs**	98.2%	98.3%	7154	82	94	41
**20 and more ESTs**	98.7%	98.8%	3154	30	33	9
**50 and more ESTs**	99.5%	99.3%	741	4	3	1

**Figure 1:**
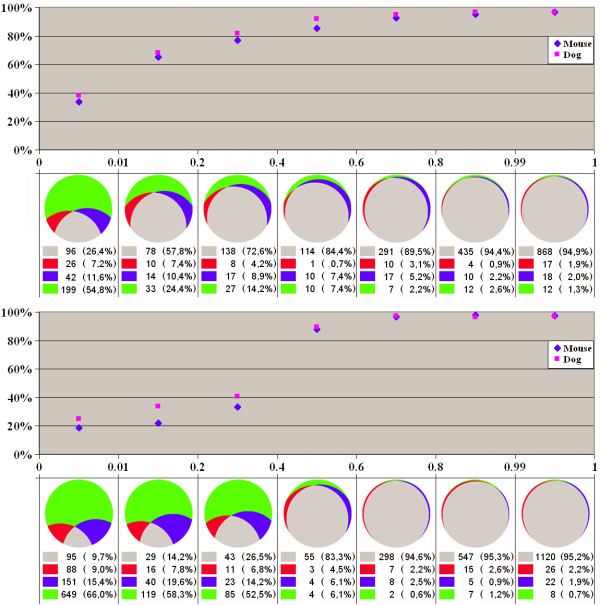
**Conservation and inclusion level of cassette exons**. Horizontal axis: inclusion level (fraction of ESTs covering an alternative region and containing the exon, see the text for detailed definitions). Top plot: Red diamonds and blue squares – percent of human exons in each bin that are conserved in the mouse and dog genomes, respectively. Crescent piecharts below: sizes of circle segments are proportional to the total number of human exons in the given bin that are conserved in both mouse and dog (grey), conserved only in mouse or dog (red and blue respectively), or human-specific (green). The percentages of these types of exons are given in the table in the middle. The leftmost bin contains exons with inclusion frequency less than 1%; the rightmost bin contains exons with skipping frequency less than 1%. Both represent possible splicing errors, see the text for details. **Top: frame-preserving exons. Bottom: frame-shifting exons**

The relationships between conservation, inclusion level, and frame-preservation of cassette exons are analyzed in Fig. 1. As is other studies, it is clear that (i) the fraction of conserved exons is higher among exons with higher inclusion level [5,9,10,19] and (ii) frame-shifting exons are conserved less often than frame-preserving exons (more generally, exons yielding potentially translated isoforms) [9]. However, the difference in the conservation fraction is really negligible for the major exons, as more than 90% of exons whose inclusion level exceeds 60% are conserved in at least one genome regardless their functionality. For the minor exons, the situation changes dramatically for frame-shifting exons, and more gradually for frame-preserving exons. However, even for very rarely included exons (skipped in more than 99% of cases), the fraction of human exons conserved in at least one other genome is approximately 40% for both frame-preserving and frame-shifting exons. However, the fraction of exons conserved in both genomes is considerably lower for the latter (10%) compared to the former (26%), and the same holds for all other inclusion levels: frame-shifting exons tend to become lost in at least one lineage. At that, an exon is more likely to be lost in the mouse genome than in the dog genome: the number of exons common for human and dog, but not mouse is about twice larger than the number of human-mouse-(not-dog) exons for all inclusion levels of both frame-preserving and frame-shifting exons. This is consistent with other evidence of faster molecular evolution in the rodent lineage compared to other mammals, and human and dog in particular [18].

**Figure 2:**
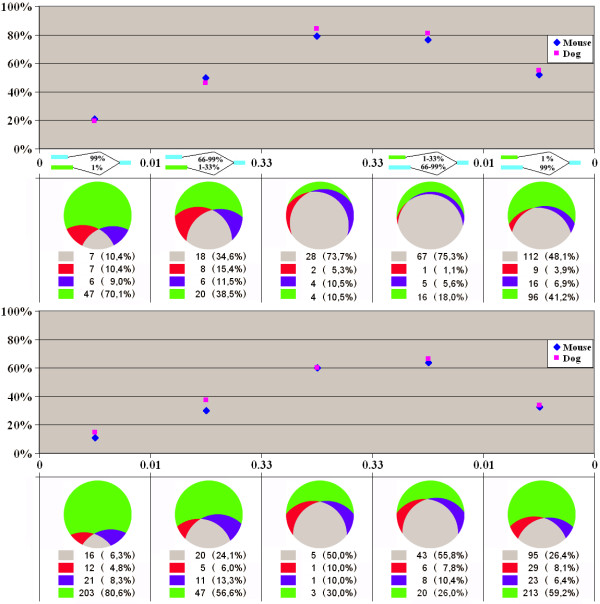
**Conservation of alternative donor splice sites**. Horizontal axis: frequencies of external and internal sites. Other notation as in Figure 1, but for sites instead of exons.

**Figure 3:**
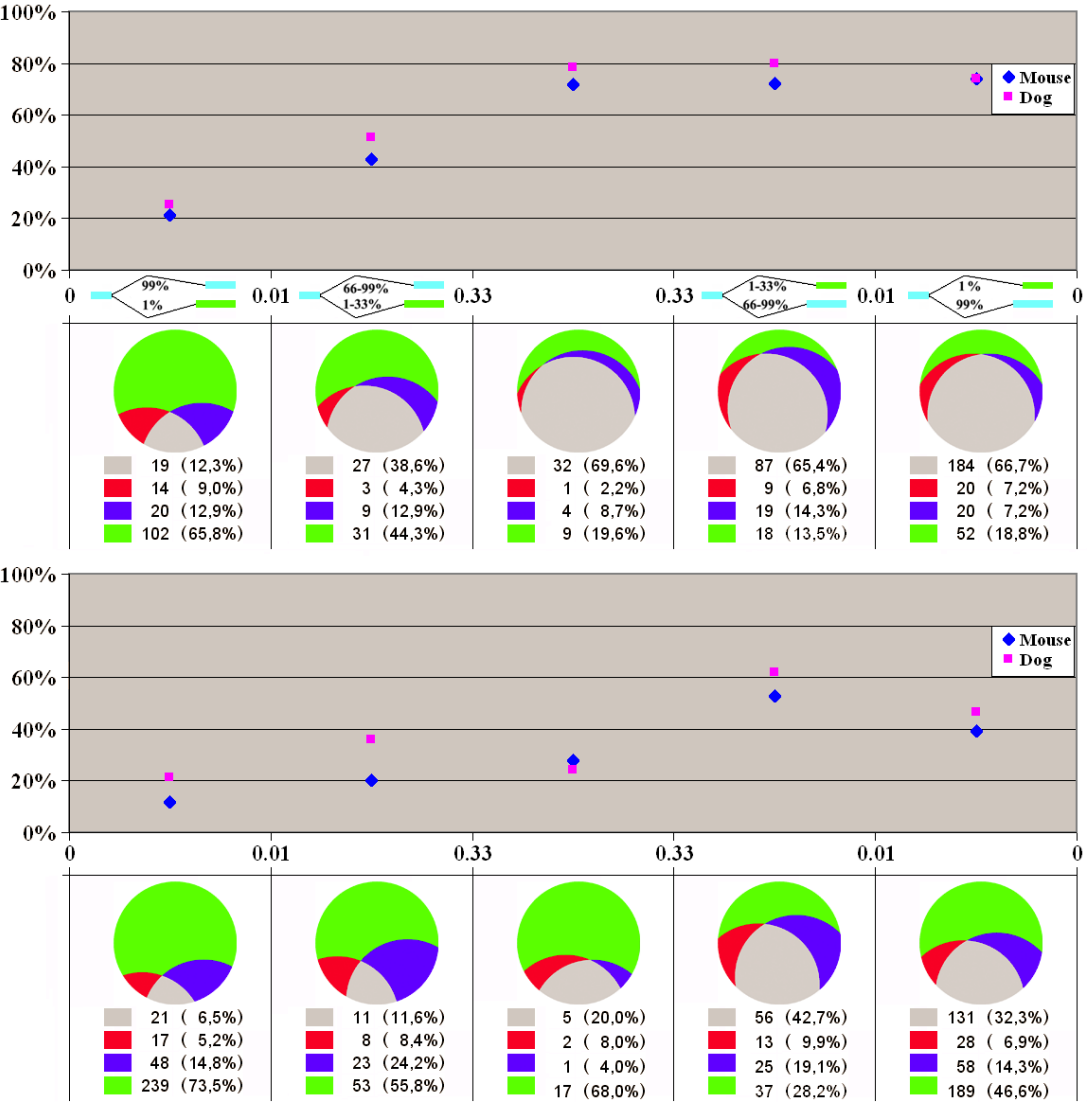
**Conservation of alternative acceptor splice sites**. Notation as in Figure 2.

A slightly more complicated situation was observed for alternative sites. Here the distinction between inclusion and exclusion transforms into the distinction between internal (yielding shorter exons) and external (yielding longer exons) sites. From the protein point of view, the use of internal sites leads to deletions (*cf. *skipped exons), whereas the use of external sites, insertions (*cf. *included exons). There exists an evolutionary asymmetry: even if an internal site does not function in splicing, it still might be conserved simply because it falls within the protein-coding region and is subject to selection acting on the level of the encoded protein, unlike an external site that in general might be expected to be conserved only if it does yield a functional isoform. As seen in Figs. 2 and 3, the conservation reaches maximum in the interval of approximately equal use of internal and external variants. As expected, the conservation of alternatives is clearly higher in the interval where frequently used sites are the external ones compared to the rarely used external sites.

The overall trends in the evolution of alternative sites are the same as in the case of cassette exons. There is a considerable level of lineage-specific loss of alternative sites, and the losses are more frequent in the mouse genome compared to the dog genome. Frame-shifting variants, both external and internal, are relatively less conserved, although many of them still are conserved in at least one genome. Uniformly used donor sites from the frame-preserving subgroup are slightly more conserved compared to acceptor sites, but both frame-shifting acceptor sites and unevenly used acceptor sites that show clear prevalence of one isoform, are more conserved than donor sites from the respective groups. However, all these differences are rather minor.

### Alternative splice sites tend to extend short introns

**Figure 4:**
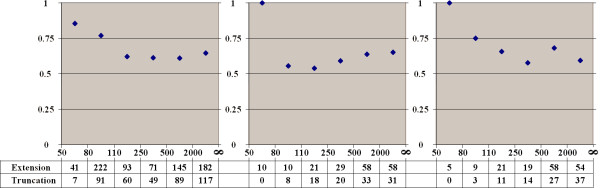
**Fraction of intron-extending donor splice sites**. Horizontal axis: intron length. Vertical axis: fraction of intron-extending donor sites among all alternative donor sites in introns of the given length. The three panels represent functional types of alternative splicing events. **Left: frame-shifting or rarely used sites. Middle: frequent, frame-preserving alternative sites. Right: frequent alternative sites conserved in either mouse or dog**

**Figure 5:**
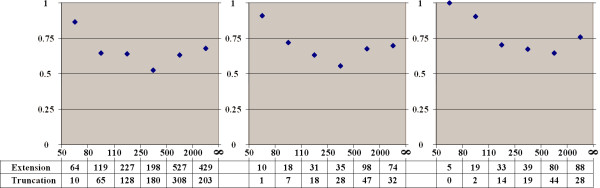
**Fraction of intron-extending acceptor splice sites**. Notation as in Figure 4.

For different intervals of intron lengths we calculated the fraction of alternative donor (Fig. 4) and alternative acceptor (Fig. 5) sites extending or truncating the intron compared to the major form. Short introns are mainly extended by alternative sites, but the fraction of intron-extending and truncating sites stabilizes at about 60% as the intron length increases.

### Distribution of alternative sites is consistent with a model of random fixation

**Figure 6:**
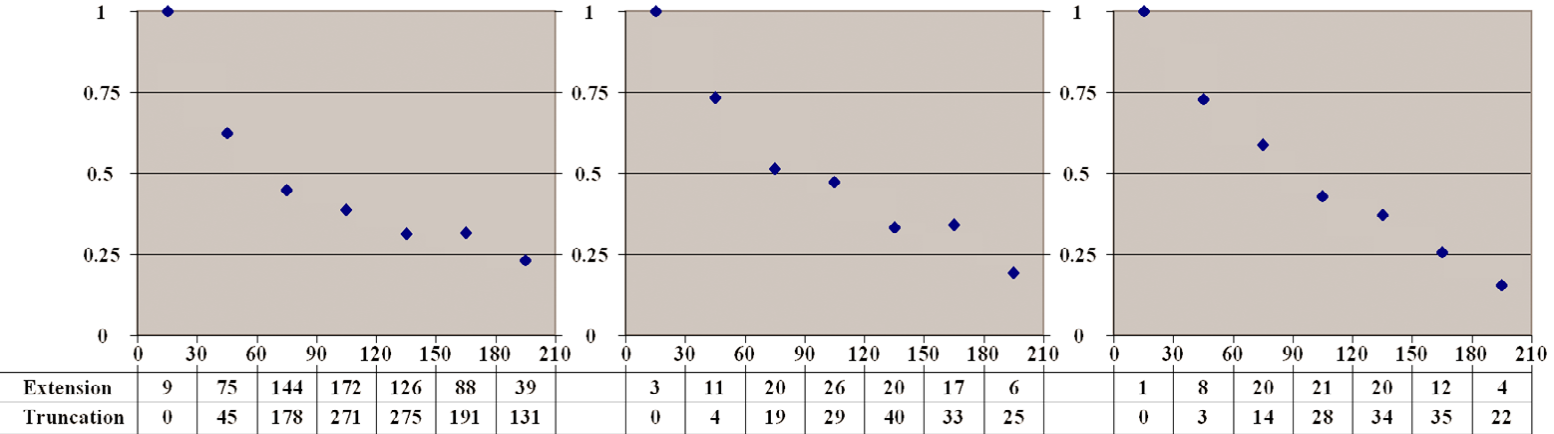
**Fraction of exon-extending donor splice sites**. Horizontal axis: exon length. Notation as in Figure 4.

**Figure 7:**
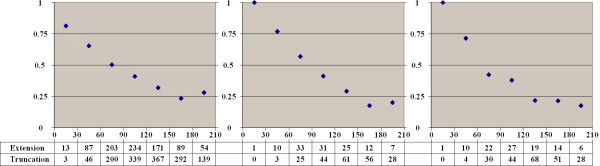
**Fraction of exon-extending acceptor splice sites**. Notation as in Figure 4.

We then analyzed the possible source of alternative sites. At that, we compared the frequencies of cases when the major site is the internal one (alternative extensions) and the external major sites (alternative truncations). The relative fraction of extensions among all alternative sites as dependent on the exon length is shown in Fig. 6 (donor sites) and Fig. 7 (acceptor sites), separately for two functional groups of alternatives sites (frequent and frame-preserving sites that are likely functional, and frame-shifting or rarely used sites that might be suspected to be non-functional). We also considered separately all alternative sites conserved in either mouse or dog. In all cases we observed a strong correlation between the tendency of alternative splice sites to be mainly extending or truncating and the exon length: alternative splice sites tend to extend short exons and truncate long ones, with the balance between the extending and truncating alternative sites reached at exons of approximately 90 nucleotides in length.

These observations are consistent with alternative splice sites arising from fixation of cryptic ones. Indeed, the probability of a cryptic site within an exon (a truncating alternative) increases with exon length. Truncation of short exons is unlikely, as there simply is no space for an alternative site. However, an alternative explanation could be that alternative sites are fixated due to selection towards preferred exon length caused by difficulties in recognition or splicing of too short or excessively long exons.

To distinguish between these possibilities, we developed a simple model of random site fixation. We assumed that the probabilities of cryptic donor and acceptor sites are the same within introns and within exons, and that only in-frame cryptic sites could be fixated as minor alternative sites.

In this case the probability of exon truncation (that is, of existence of at least one cryptic site within an exon) is roughly proportional to the exon length, whereas the probability of exon extension is proportional to the distance to the nearest in-frame stop codon in the adjacent introns. The equilibrium is reached when the probabilities of fixating a truncating cryptic site and an extending cryptic site are equal, and this happens when the exon length is twice the distance to the nearest in-frame stop codon (since an exon may be extended on both sides). When we calculated the average distance from a random point in an intron to the nearest in-frame stop codon, it was 73 nucleotides, and twice this value, 146 nucleotides, indeed is close to the average exon length that is about 130 nucleotides.

## Discussion

It has been suggested that alternative splicing serves as an evolutionary testing ground: new exons initially appear as alternative minor variants, and become constitutive following fine-tuning of regulatory elements if they prove to add new, beneficial properties to the encoded protein [5,9,10]. This is consistent with the fact that relatively rarely used isoforms are more likely to be species-specific and the evidence for faster evolution, and more positive selection in alternative regions compared to constitutive ones [21-25]. Stretching this idea a bit further, one might say that the aberrant isoforms are not simple noise, but rather raw building material, on which selection towards new functions operates.

While earlier studies [8] saw little evidence of exonisation and it was implicitly assumed that new alternative exons evolve by duplication [26,27], newer analyses indicate that exonisation may be the main source of new exons. Indeed, many studies suggest that the human genome contains a large number of cryptic sites that become activated following mutations disrupting the main sites [28,29]. New sites can also emerge as a result of activating mutations creating both alternative sites and cassette exons [30]; this is particularly true for acceptor sites where many splicing-related genetic diseases are caused by *de novo *sites [29]. A rich source of cryptic sites, both acceptor and donor, is Alu repeats [3,1,32,33]. Our analysis of extending and truncating alternative sites is consistent with the theory of fixation of randomly occurring cryptic sites. However, mutually exclusive exons indeed often evolve by duplication [26,27] and their evolutionary properties are sharply different compared to the properties of cassette exons. For example, in insects, mutually exclusive exons are as conserved as constitutive ones, and tolerate even less intron insertions than the latter [11].

When this study was essentially completed, two studies appeared that addressed the problem of exon conservation in vertebrate datasets [9,10]. Both studies mainly considered internal cassette exons and reached similar conclusions, namely, that young exons tend to be alternatively spliced and minor. At that, both studies ascribe differences between human and other genomes to the emergence of new exons. The latter study [10] used the outgroup approach to distinguish between exon birth and loss, and considered an exon present in the human genome but absent in the comparison (say, mouse) genome and the outgroup genome to be a new human exon created after branching out of the comparison genome (similarly for the exon loss). However, this approach does not guarantee that these new exons are functional and do not represent splicing errors or experimental noise [13,34]. The former study [9] used a different technique, calculating the number of exons present in the human and comparison genomes and absent in the outgroup genome; here the assumption is that such exons emerged at the branch leading to the human and comparison genomes. This is a more conservative approach, especially at larger evolutionary distances between the human and comparison genomes, since conserved exons may be assumed to be functional. However, this does not account for the possibility of exon loss in the outgroup.

Our results demonstrate that both the issue of exon functionality and the possibility of exon loss should not be ignored. Indeed, a large fraction of alternative exons and alternative sites are conserved in human and mouse but not dog or *vice versa*, which means that whatever the branching order, these differences may not be exclusively explained by lineage-specific exon birth (curiously, despite using the same Human Genome Browser genomic alignment, the cited studies assumed different branching orders, primates-rodents-dog [10] and primates-dog-rodents [9]). A high level of exon loss in rodents was also demonstrated in [17]. Thus, despite being consistent with other studies [8-10] as regards the general trends in the distribution of lineage-specific exons, our study provides evidence that lineage-specific loss of alternative exons and sites is an important factor in the evolution of alternative splicing (cf. the prevalence of intron loss over intron gain in mammals [35]). Because of that, conservation of exons should be defined not only in terms of evolutionary depth of exon presence in genomes (time of birth), but also as resistance to loss. This means that modeling of exon evolution needs a combined approach, utilizing both outgroup and ingroup techniques. This can be done not only for vertebrates, where evolution of the exon-intron structure is dominated by the exon dynamics [35], but also for (dipteran) insects, where intron insertion and loss play an important role, while the general trend of lower conservation of alternative sites and exons compared to constitutive ones is the same as in vertebrates [11]. Finally, we demonstrate that not only conservation of cassette exons depends on the exon inclusion level, but also that conservation of alternative sites depends on the relative site usage and show that both are dependent on the exon (resp. site) functionality.

## Conclusion

Our results demonstrate considerable evolutionary diversity of alternative splicing, in particular frequent lineage-specific loss of alternative variants. The fraction of conserved cassette exons is higher among exons with high inclusion level, and frame-shifting exons are less conserved than frame-preserving exons. However, the difference in the conservation level between frame-shifting and frame-preserving exons is really negligible for major exons. For very rarely included exons the fraction of human exons conserved in at least one other genome is approximately the same for both frame-preserving and frame-shifting exons, whereas the fraction of exons conserved in both genomes is considerable higher for frame-preserving compared to frame-shifting ones. For alternative splice sites the conservation reaches maximum when the internal and external variants are used approximately equally. The distribution of alternative sites is consistent with a model of random fixation: alternative splice sites tend to extend short exons, truncate long exons, and extend very short introns.

## Methods

### Construction of the sample of human elementary alterantive splicing events

All protein, mRNA, DNA and EST sequences were derived from GeneBank [36] (UniGene, EntrezGene, GenePept). EST and mRNA sequences were aligned with genomic DNA using ProEST [1], and protein sequences were aligned with genomic DNA using ProFrame [37].

**Figure 8:**
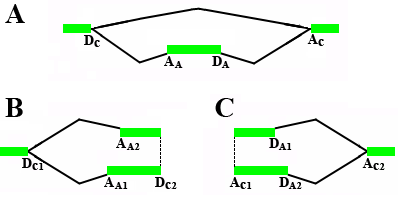
**Schematic representation of considered elementary alternatives**. Notation: D – donor sites, A – acceptor sites; subscripts: C – constant sites, A – alternative sites. **A. Cassette exons. B. Alternative acceptor sites. C. Alternative donor sites**

For each gene we constructed the splicing graph. The vertices of this graph correspond to the donor and acceptor splice sites or to the termini of first and last exons, and the directed arcs correspond to introns and exons. Then we consider pairs of sites (vertices) such that the 5'-site has at least two outcoming arcs, the 3' site has at least two incoming arcs, and there is no vertex common for all paths coming from the 5'-site to the 3'-site (note that both the 5'-site and the 3'-site may be donor sites and/or acceptor sites). Each such pair of a 5'-site and a 3'-site forms an alternative. An alternative with only two paths between the 5'-site and the 3'-site was considered elementary. Among all elementary alternatives we selected cassette exons, pairs of alternative donor sites, and pairs of alternative acceptor sites. The corresponding subgraphs are shown in Fig. 8. Retained introns, mutually exclusive exons, and complex unclassifiable alternatives were not considered.

We required that at least one path forming an alternative was supported by a protein sequence. For example, for cassette exon either the path consisting of one arc (intron D_C_-A_C_) or the path consisting of three arcs (intron D_C_-A_A_, exon A_A_-D_A_, intron D_A_-A_C_) in Fig. 8A, or both had to be observed in spliced alignments with proteins from GenePept. Thus we considered only 19350 alternatives occurring in protein-coding regions. For each alternative, the major variant was defined as the one supported by a protein sequence and having higher EST coverage. We removed 440 cases where the single protein-supported variant had lower EST coverage than the alternative, purely EST-supported variant. We also removed overlapping alternative splice sites by considering only alternative donor and acceptor sites with at least nine nucleotide positional difference. This resulted in the final sample of 18910 elementary alternatives.

Since we required that the major variant was a protein one, this procedure allowed us to set the reading frame and to distinguish between frame-preserving and frame-shifting alternative variants, as well as variants containing in-frame stop codons.

Rare exons and sites that potentially could arise from splicing errors were defined using the procedure from [19]. At that, we assumed that variants with frequency less that 1% may not be relevant. To retain only relevant variants, we excluded all cases then the hypothesis that the minor variant frequency was less than 1% could not be rejected at the 95% level. For each elementary alternative we calculated the probability that the observed minor variant counts are sampled from a distribution with the minor variant frequency of 1%:

P=∑i=0K−1N!i!×(N−i)!(0,01)i×(1−0,01)N−i
 MathType@MTEF@5@5@+=feaagaart1ev2aaatCvAUfKttLearuWrP9MDH5MBPbIqV92AaeXatLxBI9gBaebbnrfifHhDYfgasaacPC6xNi=xI8qiVKYPFjYdHaVhbbf9v8qqaqFr0xc9vqFj0dXdbba91qpepeI8k8fiI+fsY=rqGqVepae9pg0db9vqaiVgFr0xfr=xfr=xc9adbaqaaeGacaGaaiaabeqaaeqabiWaaaGcbaqaaiabdcfaqjabd2da9maaqahabaqcfa4aaSaaaeaacqWGobGtcqGGHaqiaeaacqWGPbqAcqGGHaqicqGHxdaTcqGGOaakcqWGobGtcqGHsislcqWGPbqAcqGGPaqkcqGGHaqiaaGccqGGOaakcqqGWaamcqqGSaalcqqGWaamcqqGXaqmcqqGPaqkdaahaaWcbeqaaiabdMgaPbaakiabgEna0Iqaaiab=HcaOiabbgdaXiabgkHiTiabbcdaWiabbYcaSiabbcdaWiabbgdaXiabcMcaPmaaCaaaleqabaGaemOta4KaeyOeI0IaemyAaKgaaaqaaiabdMgaPjabd2da9iabbcdaWaqaaiabdUealjabgkHiTiabbgdaXaqdcqGHris5aaaaaa@5729@

where *N *is the number of all ESTs that cover the alternative region, and *K *is the number of ESTs that correspond to the minor variant. At that, an EST was assumed to cover a cassette exon region if it covered at least partially both adjacent exons. Similarly, an EST was considered covering an alternative splice site if it covered at least partially the exon containing this site and the adjacent exon. If the probability *P *exceeded 0.95, we treated the minor alternative as a relevant one and assumed its frequency to be K/N. Otherwise the alternative was treated as a possibly spurious one.

### Testing the conservation of human elementary alternative splicing events in the mouse and dog genomes

We analyzed conservation of human exons and alternative sites in the mouse and dog genome using a two-step procedure. Firstly, we compared translated DNA sequences of human and mouse or dog genes using BLAT [38]. This allowed us to identify highly conserved human exons and split all DNA alignments into segments between such exons and, further, to localize orthologs of all human exons in the mouse and dog genomes either explicitly, or by matching of adjacent exons. Then we attempted to find orthologs of the remaining unmatched exons by genomic spliced alignment using Pro-Gene [39]. This program implements a variant of the Smith-Waterman dynamic programming algorithm. We allowed some variation at exon termini, so that one or two first or last amino acids in each human exon could be missed in the alignment. Such site shifts were forbidden for alternative donor and acceptor splice sites. This is consistent with the observation that site sliding on larger distances is rare [40].

Additionally we realigned exons that formed elementary alternatives. To analyze cassette exons, we aligned exon-intron-exon-intron-exon fragments (the central exon being the cassette exon under consideration), and an exon was assumed to be conserved if this exon and both adjacent splice sites (D_C _and A_C _in Fig 8A) were conserved. To analyze alternative sites, we considered exon-intron-exon fragments (with an alternative donor site in the left exon or an alternative acceptor site in the right exon). An alternative site was assumed to be conserved if both constitutive and alternative splice sites were conserved (pair of alternative donor sites D_A1 _and D_A2 _plus acceptor sites A_C1 _and A_C2 _in Fig. 8B; pair of alternative acceptor sites A_A1 _and A_A2_, plus donor sites D_C1 _and C_D2 _in Fig. 8C).

### Modeling of cryptic sites

To calculate the average distance to the nearest stop codon, we considered random points in constitutively spliced introns and scanned the intron in the 3' direction, storing the distance to the nearest in-frame stop codon. This procedure was repeated ten times for each constitutive intron.

## Authors' contributions

RNN, AAM and MSG conceived the project. RNN and ADN developed the splicing classification algorithm and produced the data. RNN performed statistical analysis. RNN and AVF developed the model of random sites fixation. RNN and MSG wrote the manuscript. All authors read and approved the manuscript.
